# Over-nutrition and associated factors among 20 to 49-year-old women in Uganda: evidence from the 2016 Uganda demographic health survey

**DOI:** 10.11604/pamj.2021.39.261.26730

**Published:** 2021-08-24

**Authors:** Quraish Sserwanja, David Mukunya, Joseph Kawuki, Linet Mueni Mutisya, Milton Wamboko Musaba, Ivan Kato Arinda, Mathew Kagwisagye, Shirin Ziaei

**Affiliations:** 1 Programmes Department, GOAL Global, Khartoum, Sudan,; 2 Department of Public Health, Busitema University, Tororo, Uganda,; 3 Sanyu Africa Research Institute, Mbale, Uganda,; 4 Centre for Health Behaviours Research, Jockey Club School of Public Health and Primary Care, The Chinese University of Hong Kong, Hong Kong, China,; 5 Maternal and Child Health Project, Swedish Organization for Global Health, Mayuge, Uganda,; 6 Department of Obstetrics and Gynecology, Busitema University, Tororo, Uganda,; 7 Department of Obstetrics and Gynaecology, Makerere University College of Health Sciences, Kampala, Uganda,; 8 School of Public Health, Makerere University College of Health Sciences, Kampala, Uganda,; 9 Department of Women´s and Children's Health, Uppsala University, Uppsala, Sweden

**Keywords:** Overweight, obesity, over-nutrition, prevalence, women, Uganda

## Abstract

**Introduction:**

low- and middle-income countries are currently faced with a double burden of malnutrition. There has, however, been little focus on research and interventions for women with over-nutrition. We aimed to determine the prevalence and factors associated with over-nutrition among 20 to 49-year-old women in Uganda.

**Methods:**

we used the Uganda demographic and health survey (UDHS) 2016 data of 4,640 women. We analysed data using SPSS (version 25), and we used multivariable logistic regression to determine factors associated with over-nutrition among 20 to 49-year-old women in Uganda.

**Results:**

the prevalence of over-nutrition was 28.2% (95% confidence interval (CI): 26.8-29.4) with overweight at 19.3% and obesity at 8.9%. Women belonging to the poorer (adjusted odds ratio (AOR)=1.63; 95% CI: 1.17-2.28), middle (AOR=2.24; 95% CI: 1.61-3.13), richer (AOR=3.02; 95% CI: 2.14-4.25) and richest (AOR=6.35; 95% CI: 4.52-8.93) wealth index quintiles were more likely to be over-nourished compared to women in the poorest wealth index quintile. Married women (AOR=1.52; 95% CI: 1.26-1.83) were more likely to be over-nourished compared to non-married women. Older women were more likely to be over-nourished compared to younger women. Women in the Western (AOR=2.12; 95% CI: 1.66-2.71), Eastern (AOR=1.40; 95% CI: 1.04-1.88) and Central (AOR=2.25; 95% CI: 1.69-2.99) regions were more likely to be over-nourished compared to women in the Northern region.

**Conclusion:**

the design of multi-faceted over-nutrition reduction programs with an emphasis on older, married, financially stable women, and those living in the Western, Eastern and Central regions of the country is needed.

## Introduction

Over-nutrition is a form of malnutrition arising from a general imbalance in energy intake compared to energy expenditure [1] and is regarded as one of the major causes of mortality globally [2]. Overweight and obesity are indicators of over-nutrition, and an adult is considered to be overweight when their body mass index (BMI) is between 25 and 29.99 kg/m^2^ and considered obese when it is above 29.99 kg/m^2^ [3,4]. Over-nutrition has steadily increased since 1980 [5] with current global adult overweight prevalence at 39% (39% men and 40% women) while 13% are obese (11% men and 15% women) [6]. Despite the high burden of underweight among low-income countries, they are currently faced with increasing rates of over-nutrition [3]. Amugsi *et al*. analyzed data from 24 African countries over 23 years and showed increasing prevalence of over-nutrition [7].

Maternal over-nutrition has been associated with negative outcomes including gestational diabetes, pre-eclampsia, an increased miscarriage rate, still-births and congenital anomalies as well as higher risk of obesity among their children in later life [7,8]. Furthermore, over-nutrition has been shown to decrease contraceptive efficacy and to increase the risk of ovulatory disorders [7]. Maternal over-nutrition has also been shown to negatively affect the children´s feeding practices as overweight women have been shown to be more likely to stop breastfeeding when the infant shows satiation cues and have been reported to use more restrictive feeding practices [7,9]. The rising prevalence of over-nutrition further increases the risk of non-communicable diseases (NCDs), such as cardiovascular disease, diabetes, musculoskeletal disorders and cancers [6].

Low and middle-income countries have historically experienced high levels of under-nutrition [10,11], and recently, the observed rise in the prevalence of over-nutrition has led to a double burden of malnutrition [3]. This has presented challenges for public healthcare systems in these countries as they mainly devote their resources to problems of under-nutrition and infectious diseases but now have to deal with over-nutrition related diseases [3,12]. Despite the recent evidence of increased over-nutrition, and recognition of the potential rise in over-nutrition-associated NCDs, in low and middle-countries, little effort has been made in addressing over-nutrition [7]. Most studies in Uganda and public health interventions have mainly focused on under-nutrition with little focus on research and interventions that target women with over-nutrition [13-15]. Planning of effective interventions for women with over-nutrition requires an identification of the prevalence and risk factors for over-nutrition hence we aimed to determine the prevalence and factors associated with over-nutrition among 20 to 49-year-old women in Uganda.

## Methods

**Study data:** we conducted a secondary analysis of the nationally-representative 2016 Uganda demographic health survey (UDHS) data collected from June 2016 to December 2016 [16]. The survey was household-based, implemented by the Uganda Bureau of Statistics (UBOS) with the technical assistance of the Inner City Fund (ICF) International through the The United States Agency for International Development (USAID)-supported Monitoring and Evaluation to Assess and Use Results (MEASURE) DHS project [16]. The survey obtained detailed health and demographic information using the household questionnaire, women´s questionnaire, men´s questionnaire and biomarker questionnaire collected data on anthropometry and blood tests [16]. Our study used data from the women´s questionnaire, which collected information about women´s characteristics, their reproductive health history and their homes [16]. High international ethical standards are ensured during MEASURE DHS surveys as ethical approval from the country is obtained from a national ethical review board. Besides, the local authorities’ approval before implementing the survey and respondents' well-informed verbal consent are sought prior to data collection [16]. This data set was obtained from the MEASURE DHS website after getting their permission, and no formal ethical clearance was obtained since we conducted a secondary analysis of publicly available data.

**Study setting:** according to the 2019/2020 Uganda National Health Survey, Uganda has a population of 41 million people with an average of 4.6 persons per household, 3.5% prevalence of non-communicable diseases and 27% of the population residing in urban areas [17]. Uganda´s health system has six levels ranging from the highest level of national referral hospitals to the lowest level at the community level [18]. Agriculture contributes about 24% of gross domestic product (GDP), providing half of export earnings and is the main source of income for 68% of Ugandans [17,19].

**Figure 1:**
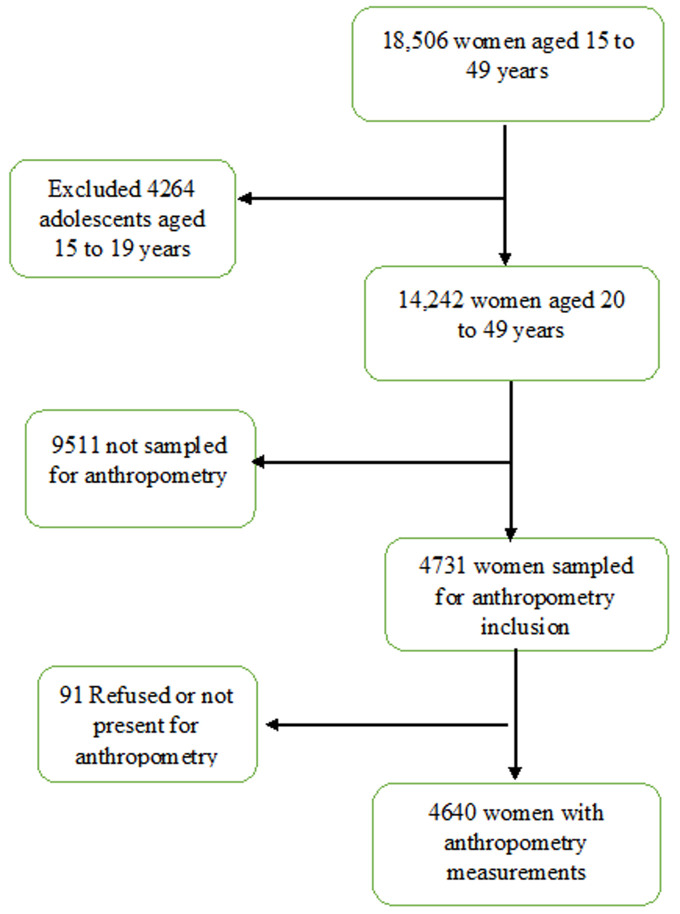
flow chat of sampling process

**Study sampling and participants:** DHS employed a two-stage cluster sampling technique where the census enumeration areas were the primary sampling units while households comprised the second stage of sampling [16]. The enumeration areas were selected from the 2014 population and housing census sample frame [16]. Women aged 15 to 49 years who were either the permanent residents or slept in the selected household the night before were eligible to be interviewed [16]. During the survey, anthropometric measurements were done for women who were not pregnant or had no birth two months before the survey. Our secondary analysis only considered 20 to 49-year-old women and excluded 15 to 19-year-old women (adolescents) because the recommended anthropometric indicators for assessing over-nutrition for those above 20 are different from those of adolescents) [20,21]. Of the 18,506 women who consented and filled in the questionnaires, 14,242 were aged 20 to 49 years and of these, 4,731 were eligible for anthropometry, 91 had missing anthropometry data and hence, a final sample of 4,640 women with complete anthropometry data were included in the analysis. Sampling process flow chart is included as Figure 1.

### Variables

**Dependent variables:** women´s nutritional status was measured by body mass index (BMI). BMI defined as weight in kilograms divided by height in meters squared (kg/m^2^) was used to measure overweight and obesity. Weight was recorded in kilograms to the nearest one decimal point and was measured using an electronic scale (SECA 878) while height was recorded in centimetres to one decimal point [16]. A BMI of above 24.99 kg/m^2^ was used as the cut-off for overweight while BMI above 29.99 kg/m^2^ was used to indicate obesity [3]. Overweight and obesity were combined in this analysis and used to define over-nutrition as done in by Doku *et al*. in Ghana [2].

**Independent variables:** this study included determinants of over-nutrition based on evidence from available literature and data [3,6,22]. These factors were divided into the individual level (age, marital status, working status and education level), household level (wealth index, household size and sex of household head) and community level (region and residence) characteristics. Wealth index was a measure of relative household economic status and was calculated by DHS from information on household asset ownership using principal component analysis and further categorized into quintiles (poorest, poorer, middle, richer and richest) at the national level [16].

Place of residence was aggregated as urban and rural. The region was categorized into four; Northern, Central, Eastern and Western. Level of education was categorized into: no education, primary education, secondary and higher education. Age was categorized into 20-29, 30-39 and 40-49. Household size was categorized as less than six members and six and above members. Sex of household head was categorized as male or female. Working status was categorized as: not working and working. Marital status was categorized into married (and this included those in formal and informal unions) and not married.

**Statistical analysis:** complex sample analysis was performed using SPSS (version 25.0) statistical software to account for the multi-stage cluster study design. The analysis was carried out based on the weighted count to account for the unequal probability sampling in different strata [22] and to ensure the representativeness of the survey results at the national and regional level. Frequencies and proportions were computed for the categorical data. Independent variables were cross-tabulated by the outcome categories, and Chi-square values were used to test for significant associations. Bivariable logistic regressions were done separately for each independent variable and crude odds ratios (CORs) were created (unadjusted model I). Independent variables found significant at p-value less than 0.2 were regressed together in a multivariable logistic regression (adjusted model II) to identify the adjusted effect of each factor on over-nutrition. Adjusted odds ratios (AOR), 95% confidence intervals (CI) and p-values were calculated with the statistical significance level set at p-value <0.05. Tests for collinearity between covariates were performed with a cut off value of above 10 variance inflation factor being used [3].

**Ethics approval and consent to participate:** high international ethical standards are ensured for MEASURE DHS surveys as ethical approval from the country is obtained from a national ethical review board and local authorities before implementing the survey and well-informed verbal consent is sought from the respondents prior to data collection [16,23]. This data set was obtained from the MEASURE DHS website after getting their permission and no formal ethical clearance was obtained since we conducted secondary analysis of publicly available data.

## Results

A total of 4,640 women were included in this study (Table 1). Almost three-quarters of the women resided in rural areas (73.6%), were currently working (84.3%) and married (73.4%). Besides, over half of the women lived in households with less than six members (53.9%), had primary education as the highest level (55.9%) and resided in male-headed households (64.5%). Regarding geographical location, the central region had the highest proportion of women (30.2%) while Eastern had the lowest (19.7%). Almost half of the women (48%) were aged 20 to 29 years. The richest wealth quintile had the highest proportion of women (25.7%) with the poorest and poorer indices having the lowest proportion (17.6% each). The mean age, weight, height, household size and BMI were 31 ± 8.18, 59.5 ± 11.86, 158.9 ± 6.37, 5.7 ± 00, and 23.56 ± 4.45 respectively. The prevalence of over-nutrition was 28.2% (95% CI: 26.8-29.4) with overweight at 19.3% and obesity at 8.9%.

**Table 1:** background characteristics of Ugandan women aged 20 to 49 years as per the 2016 UDHS

Characteristics	N=4640	%
**Age**		
20 to 29	2225	48.0
30 to 39	1486	32.0
40 to 49	928	20.0
**Residence**		
Urban	1224	26.4
Rural	3416	73.6
**Region**		
Western	1182	25.5
Eastern	913	19.7
Central	1400	30.2
Northern	1144	24.7
**Sex household head**		
Female	1648	35.5
Male	2992	64.5
**Household size**		
6 and above	2138	46.1
Less than 6	2502	53.9
**Working status^a^**		
Not working	721	15.6
Working	3913	84.4
**Marital status**		
Married	3406	73.4
Not married	1234	26.6
**Education level**		
No education	555	12.0
Primary education	2593	55.9
Secondary education	1085	23.4
Higher	407	08.7
**Wealth index**		
Poorest	816	17.6
Poorer	815	17.6
Middle	871	18.8
Richer	943	20.3
Richest	1195	25.7
**Over-nutrition**		
Obesity	414	8.9
Overweight	893	19.3
No	3333	71.8


a Missing 6 participants (0.13%)

**Factors associated with over-nutrition:** cross-tabulation results are shown in Table 2. Factors associated with over-nutrition were: wealth index, marital status, age and region, as indicated in Table 3. Women belonging to the richest (AOR=6.35; 95% CI: 4.52-8.93), richer (AOR=3.02; 95% CI: 2.14 - 4.25), middle (AOR=2.24; 95% CI: 1.61 - 3.13) and poorer (AOR=1.63; 95% CI: 1.17 - 2.28) wealth index quintiles were 535%, 202%, 124% and 63% more likely respectively to have over-nutrition compared to those in the poorest wealth index quintile. Married women were 52% more likely to be over-nourished compared to non-married women (AOR=1.52; 95% CI: 1.26-1.83).

**Table 2:** distribution of over nutrition by sociodemographic characteristics among Ugandan women aged 20 to 49 years

Characteristics	Over-nourished	Not over-nourished	P-Value
**Household head**			0.698
Female	470 (36.0)	1178(35.4)	
Male	837 (64.0)	2154(64.6)	
**Wealth index**			**<0.001**
Poorest	77 (5.9)	739 (22.2)	
Poorer	138 (10.6)	677 (20.3)	
Middle	215 (16.4)	656 (19.7)	
Richer	290 (22.2)	653 (19.6)	
Richest	588 (45.0)	607 (18.2)	
**Working status**			0.974
Working	1104(84.5)	2809(84.4)	
Not working	203 (15.5)	518 (15.6)	
**Education level**			**<0.001**
No education	127 (9.7)	428 (12.8)	
Primary	626 (47.9)	1966(59.0)	
Secondary	368 (28.2)	717 (21.5)	
Higher	186 (14.2)	221 (6.6)	
**Region**			**<0.001**
Western	367 (28.1)	815 (24.5)	
Eastern	201 (15.4)	712 (21.4)	
Central	583 (44.6)	818 (24.5)	
Northern	157 (12.0)	987 (29.6)	
**Marital status**			**0.041**
Married	987 (75.5)	2418(72.6)	
Not married	320 (24.5)	914 (27.4)	
**Age**			**<0.001**
20 to 29	514 (39.3)	1711(51.4)	
30 to 39	479 (36.6)	1007(30.2)	
40 to 49	314 (24.1)	614 (18.4)	
**Residence**			**<0.001**
Rural	800 (61.2)	2616(78.5)	
Urban	507 (38.8)	716 (21.5)	
**Household size**			0.089
Six and above	577 (44.1)	1562(46.9)	
Less than 6	731 (55.9)	1770(53.1)	

**Table 3:** determinants of over-nutrition among Ugandan women aged 20 to 49 years

Characteristics	Crude model (n=4640) COR (95%CI)	Adjusted odds ratio (n=4640) AOR (95%CI)	p-value
**Age**			
20 to 29	1	1	**<0.001**
30 to 39	1.58 (1.34-1.88)	1.78 (1.46-2.16) *	
40 to 49	1.71 (1.41-2.06)	2.26 (1.84-2.78) *	
**Education level**			
No education	1	1	0.113
Primary	1.07 (0.85-1.36)	1.41 (0.95-2.10)	
Secondary	1.73 (1.29-2.33)	1.02 (0.74-1.45)	
Higher	2.84 (2.01-4.02)	1.02 (0.79-1.31)	
**Marital status**			**<0.001**
Not married	1	1	
Married	1.17 (0.97-1.40)	1.52 (1.26-1.83) *	
**Region**			
Northern	1	1	**<0.001**
Western	2.84 (2.19-3.68)	2.12 (1.66-2.71) *	
Eastern	1.78 (1.32-2.40)	1.40 (1.04-1.88) *	
Central	4.50 (3.42-5.91)	2.25 (1.69-2.99) *	
**Household size**			
Less than 6	1	1	0.808
Six and above	0.89 (0.76-1.06)	0.98 (0.81-1.18)	
Wealth index			
Poorest	1	1	
Poorer	1.95 (1.41-2.71)	1.63 (1.17-2.28) *	**<0.001**
Middle	3.14 (2.30-4.29)	2.24 (1.61-3.13) *	
Richer	4.26 (3.09-5.87)	3.02 (2.14-4.25) *	
Richest	9.32 (6.99-12.41)	6.35 (4.52-8.93) *	
**Residence**			
Urban	1	1	0.701
Rural	0.43 (0.36-0.52)	0.95 (0.74-1.23)	

* : significant at p-value <0.05; final model - adjusted for residence, region, age, household size, marital status, education level and wealth index; AOR: adjusted odds ratio; COR: crude odds ratio

Women aged 40 to 49 years (AOR=2.26; 95% CI: 1.84-2.78) and 30 to 39 years (AOR=1.78; 95% CI: 1.46-2.16) were 126% and 78% more likely respectively to be over-nourished compared to their younger counterparts aged 20 to 29 years. Women in the Western (AOR=2.12; 95% CI: 1.66-2.71), Eastern (AOR=1.40; 95% CI: 1.04-1.88) and Central (AOR=2.25; 95% CI: 1.69-2.99) regions were 112%, 40% and 125% more likely respectively to have over-nutrition compared to those in the Northern region.

## Discussion

Prevalence of over-nutrition was 28.2% with overweight at 19.3% and obesity at 8.9%. Our study prevalence is lower than the global women overweight prevalence of 40% and obesity prevalence of 15% [6] and it is lower than the prevalence reported in high-income countries such as the United States of America [24]. The lower prevalence observed in our study can be attributed to the increased physical activity resulting mainly from less sedentary travel and work-related activities and less consumption of energy-dense foods compared to high-income countries [1,25,26]. According to a previous national survey, 94.3% of Ugandans meet the World Health Organization (WHO) physical activity recommendations, and this is mainly achieved through travel and work-related activities of moderate-intensity [27]. However, the prevalence of over-nutrition among 15-49-year-old women in Uganda has steadily increased over time from 14% in 2001 to 24% in 2016 [16]. This increasing trend of over-nutrition is worrying, and the economic growth that has led to diet and lifestyle changes could partly explain this trend [1,28].

The prevalence proportion in this study was close to that of the pooled prevalence shown by Amugsi *et al*. from 24 African countries´ DHS data [7]. However, our prevalence was higher than those in Ethiopia [3,22] and in a study that looked at women in 32 African countries [29] but lower than that in Zimbabwe [6]. Tebekaw *et al*. Abrha *et al*. and Neupane *et al*. used DHS data that was collected earlier than our data (2011 and 2013) which can explain the lower prevalence than our study since over-nutrition has been shown globally and in Africa to increase over time. The differences in the dietary patterns, lifestyles and levels of urbanization/economic development among these countries could also explain the observed differences. Wealth index, marital status, age and region were positively associated with over-nutrition.

The probability of being over-nourished increased with increasing wealth index quintiles, with women in the richest wealth index having the highest risk of over-nutrition. The rapid urbanization in Uganda [30] is contributing to more sedentary lifestyles with little physical activity. Most of these byproducts of urbanization such as motorized transport, an advanced technology at work, and expensive energy-dense foods are mostly available and accessible to wealthier women [31,32]. In a study conducted in Kampala, Uganda, wealthier women were more likely to consume energy-dense fast-foods [33], yet increased accessibility and consumption of energy-dense foods is a risk factor for over-nutrition. Furthermore, in most African communities, the larger body size is associated with being wealthy and the societal pressure forces women to gain more weight in order to keep up the wealthy appearance [6]. Wealth status has also been shown to be associated with over-nutrition in other studies [2,6,12,22].

Married women were more likely to have over-nutrition compared to the unmarried. This can be attributed to the dietary pattern changes after marriage and increased social support [34]. Married women usually have more stable eating patterns [34] and have been shown to have increased frequency of meals compared to when they are living alone [20,35]. This could be attributed to the fact that they have easier access to food due to the increased social support and the responsibility of eating together unlike the unmarried women who are more likely to skip meals [34]. Married women are also less likely to engage in physical exercises due to limited time, given the increased domestic chores after working hours [36]. With increased food frequency, coupled with less physical activity, married women are prone to being over-nourished. Some African social and cultural norms view beauty in terms of larger body size, which might lead to women with larger body size being easily married off hence the observed effect [20]. Marital status has been shown to be associated with over-nutrition in Ethiopia [22], Ghana [2] and Zimbabwe [6].

Older women were more likely to be over-nourished compared to younger women. Older age has been shown to be associated with an increase in adipose tissue, a decrease in the level of physical activity and a higher intake of energy-dense foods [22,37]. Age as a determinant of over-nutrition has also been shown in similar studies done in Ethiopia [3,22] and Ghana [2].

Region was one of the factors shown in this study to be associated with over-nutrition. In our study women residing in Western, Eastern and Central regions were more likely to have over-nutrition compared to those from the Northern region. The association was greatest in Central region followed by Western and then Eastern. Uganda has experienced a high rate of economic development and urbanization which is concentrated mainly in the Central region which also happens to be the central business district and location of the capital city [30,38]. Evidence shows that the Central region has the highest GDP per capita, followed by the Western region with the Northern region, having some of the poorest districts in Uganda [38]. As a result of this development, women in the Central and Western regions might have better access to improved modes of transport, employment in the formal sector with improved technologies, enough household resources leading to diversification of diet by including high-calorie foods and more sedentary lifestyles.

Consumption of fast-food in Uganda has greatly increased mainly in developed urban areas due to the limited time available for the working-class people in these developed urban regions which forces them to dine out rather than preparing meals at home [33]. This was also evidenced by Ayo´s study that looked at fast-food consumption in Kampala, Central Uganda and showed that 90% of the respondents consumed fast-food [33]. Furthermore, the Central and Western regions receive higher amounts of rainfall and produce more crop yields which have led to a higher level of food security [39]. The increased food availability, consumption of fast-foods and increase in sedentary lifestyles predispose these women to over-nutrition. Region as a determinant of over-nutrition has also been shown in other studies [12,22].

**Strengths:** we used a nationally representative sample and weighed the data for analysis, and therefore our results are generalized to all Ugandan women aged 20 to 49 years. Standardized procedures are a requirement of DHS surveys in data collection and validated questionnaires are used, which ensures the internal and external validity of the results.

**Limitations:** the cross-sectional design is limited by lack of temporality; hence causality inferences cannot be made. Most data on the predictors were based on self-reporting and could not be verified through records and hence a possibility of information bias. Other significant predictors of over-nutrition such as physical activity and dietary intake, were not included in the analysis as they were not available.

## Conclusion

The prevalence of over-nutrition (overweight and obesity) in our study was found to be lower than the global adult average but significant for public health consideration. Wealth, marital status, age and region were associated with over-nutrition in this study. Strategies and policies that place particular emphasis on older and richer women, and those living in the Western, Eastern and Central regions of Uganda are needed. More studies are also needed to assess for significant predictors, including the level of physical activity and nutritional history.

### What is known about this topic

- Low and middle-income countries are currently faced with a double burden of malnutrition;
- Over-nutrition is regarded as one of the major causes of mortality globally;
- Over-nutrition has steadily increased with current global adult overweight prevalence at 39%.

### What this study adds

- The prevalence of over-nutrition (28.2%) among Ugandan women using the most recent UDHS data;
- Wealth, marital status, age and region being factors that are associated with over-nutrition among Ugandan women;
- Need to focus on older and richer women, and those living in the Western, Eastern and Central regions as these are more likely to have over-nutrition.
